# First-Principles Perspective on Gas Adsorption by [Fe_4_S_4_]-Based Metal–Organic Frameworks

**DOI:** 10.1021/acs.langmuir.2c02609

**Published:** 2022-12-29

**Authors:** Fatemeh Keshavarz, Nima Rezaei, Bernardo Barbiellini

**Affiliations:** † Department of Physics, School of Engineering Science, LUT University, Yliopistonkatu 34, FI-53850 Lappeenranta, Finland; ‡ Department of Separation Science, School of Engineering Science, LUT University, Yliopistonkatu 34, FI-53850 Lappeenranta, Finland; § Department of Physics, Northeastern University, Boston, Massachusetts 02115, United States

## Abstract

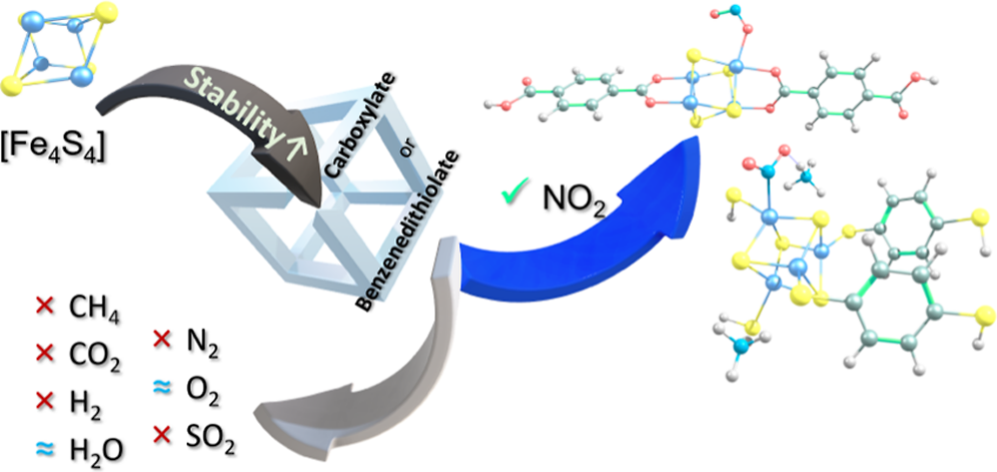

[Fe_4_S_4_] or [4S–4Fe] clusters are responsible for storing and transferring electrons in key cellular processes and interact with their microenvironment to modulate their oxidation and magnetic states. Therefore, these clusters are ideal for the metal node of chemically and electromagnetically tunable metal–organic frameworks (MOFs). To examine the adsorption-based applications of [Fe_4_S_4_]-based MOFs, we used density functional theory calculations and studied the adsorption of CO_2_, CH_4_, H_2_O, H_2_, N_2_, NO_2_, O_2_, and SO_2_ onto [Fe_4_S_4_]^0^, [Fe_4_S_4_]^2+^, and two 1D MOF models with the carboxylate and 1,4-benzenedithiolate organic linkers. Our reaction kinetics and thermodynamics results indicated that MOF formation promotes the oxidative and hydrolytic stability of the [Fe_4_S_4_] clusters but decreases their adsorption efficiency. Our study suggests the potential industrial applications of these [Fe_4_S_4_]-based MOFs because of their limited capacity to adsorb CO_2_, CH_4_, H_2_O, H_2_, N_2_, O_2_, and SO_2_ and high selectivity for NO_2_ adsorption.

## Introduction

Metal–organic frameworks (MOFs) are highly crystalline and porous materials made of metal nodes and organic ligands. The composition of MOFs can be tailored to achieve the desired physiochemical properties for specific applications.^1^ This flexibility has triggered the fast development of various MOFs and their applications, particularly in adsorption-based processes, including carbon capture, catalysis, gas storage, sensors, drug delivery, and wastewater treatment.^2−4^ Sometimes the chemical modification alone is not enough, and the MOF should be responsive to other stimuli, for example, thermal treatment, magnetic fields, and electric/redox modulation to obtain the optimal efficiency.^5−8^ Therefore, the application of thermally and magnetoelectric-responsive metal nodes in MOFs can extend their industrial applications. Despite these promising features, the application of [Fe_4_S_4_] or [4S–4Fe] clusters as the MOF metal sites is overlooked in the literature. Along with other Fe–S clusters, these clusters are the building blocks of many metalloenzymes and metalloproteins, such as nitrogenase, hydrogenase, and ferredoxins.^9,10^ Through electron transport, these Fe–S clusters facilitate DNA repair, catalytic transformations, cellular respiration, vitamin biosynthesis, and photosynthesis.^11−13^ Their electron transfer ability relies on the redox reactions of their Fe atoms.^10,13^

**Figure 1:**
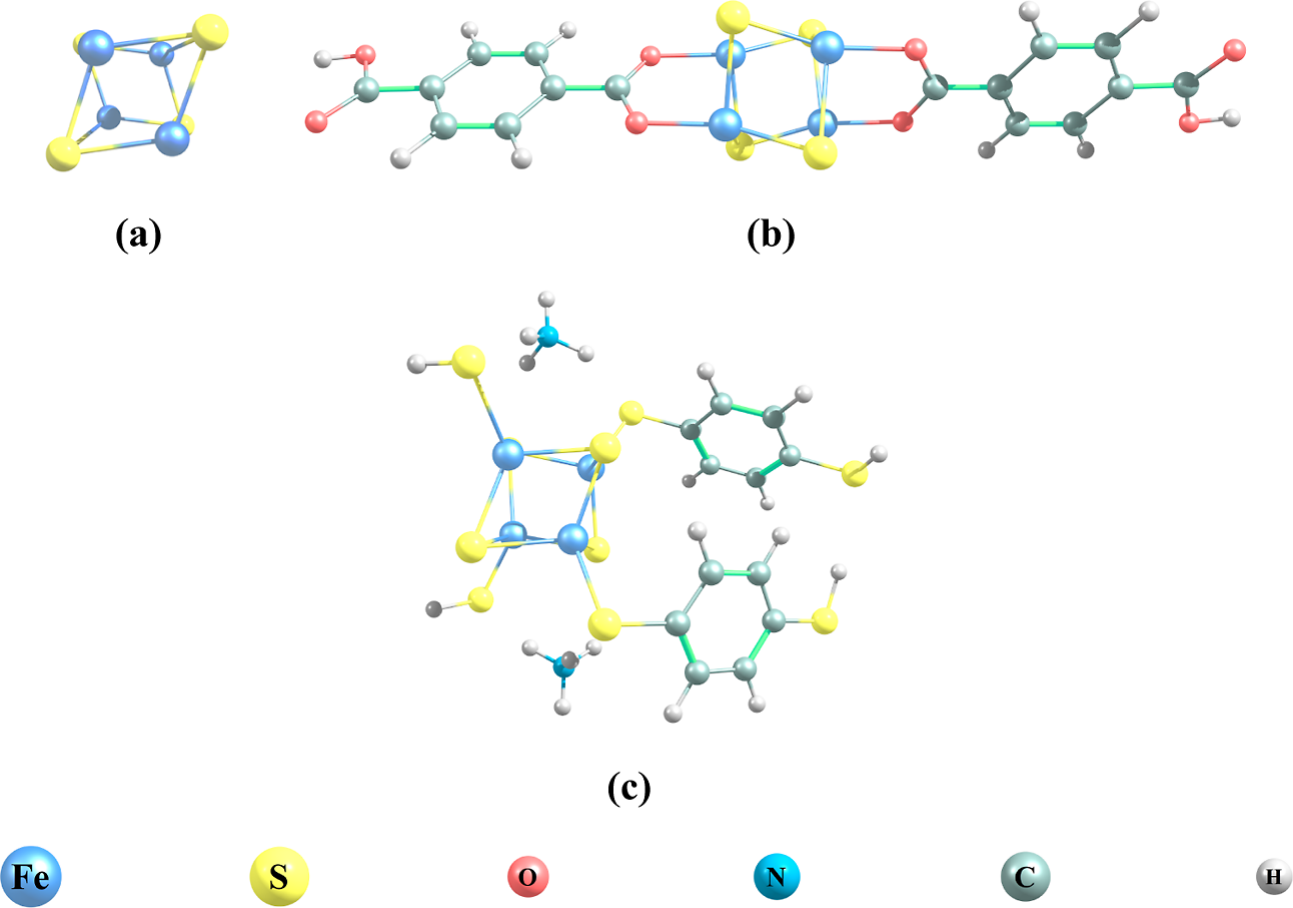
Molecular structures of the studied stand-alone clusters (CLN and CLP) (a) and the carboxylate (CMOF) (b) and 1,4-benzenedithiolate (BMOF) (c) containing MOF models.

The properties of [Fe_4_S_4_] clusters are highly affected by their microenvironment,^10,11,14,15^ and their spin-regulation and superexchange interactions modulate their long-range electron transfer properties;^16^ therefore, carefully designed [Fe_4_S_4_]-based MOFs are expected to provide bio-mimicking features that are advantageous in industrial applications.^17^ For instance, Horwitz et al.^18^ have showed that a redox-active 1D coordination polymer (or 1D-MOF) made of 1,4-benzenedithiolate ligands and [Fe_4_S_4_]^2+^ metal nodes features a high electrical conductivity. To provide some insight into the prospective applications of the [Fe_4_S_4_]-based MOFs in gas adsorption, we evaluated the performance of the reduced [Fe_4_S_4_]^0^ cluster (CLN) and highly oxidized [Fe_4_S_4_]^2+^ cluster (CLP) and also two simple 1D-MOF models containing highly stable [Fe_4_S_4_]^2+^ metal nodes with carboxylate (CMOF) and 1,4-benzenedithiolate (BMOF) ligands (see Figure 1). For BMOF, the orientation of the organic linkers was adjusted to represent that of the periodic MOF structure reported by Horwitz et al.^18^ For CMOF, the only possible ligand orientation was considered. Also, we studied the oxidative and hydrolytic stability of all adsorbents. The selected adsorbate gases included CO_2_, CH_4_, H_2_, H_2_O, N_2_, NO_2_, O_2_, and SO_2_, which are of significant importance in chemical and environmental engineering applications. The selected carboxylate ligand is the most conventional MOF linker,^19^ and the [Fe_4_S_4_]^0^ and [Fe_4_S_4_]^2+^ clusters represent two important oxidation states.^20^

## Methods

The thermodynamics and kinetics evaluations were based on first-principles, using density functional theory calculations by the Gaussian 16 A.03 package^21^ at the PBE/6-311++g(d,p)^22^ computational level and by considering the D3 Grimme’s dispersion correction.^23^Section S1 in the Supporting Information outlines the computational details on the selection of the spin states, computational level calibration (Tables S1–S4), thermodynamics studies, and the reaction mechanism explorations. Despite our careful computational level calibration and ground-state modulations, the accuracy of our results should be only considered acceptable qualitatively. We cannot confirm the quantitative accuracy of our thermodynamics and kinetics results because of lack of experimental data. We expect the results to be affected by inaccuracy and uncertainty to some extent as large active space calculations and dynamical correlation inclusion are required to accurately describe the challenging electronic structure of some [Fe_4_S_4_] clusters, for example, [Fe_4_S_4_(SMe)_4_]^−2^.^24^

## Results and Discussion

Tables 1 and S4–S6, respectively, report the adsorption Gibbs free energy, electronic energy, enthalpy, and entropy values for various adsorption modes, and Figure S1 shows the most favorable adsorbate/adsorbent configurations (identified with the lowest energy). According to the thermodynamics results, all four adsorbents can inherently adsorb all examined gases (Table S5). However, their performances are affected by the change of temperature and the inclusion of thermal effects. There are no significant differences between the enthalpy (Δ*H*) (Table S6) and electronic energy (Δ*E*) (Table S5) values, meaning that the thermal contribution to the adsorption enthalpy is not considerable for the studied adsorbate–adsorbent pairs. However, the decrease of entropy (Δ*S*) (Table S7) increases the Gibbs free energy of adsorption (Δ*G* = Δ*H* – *T*Δ*S*) at temperature *T* = 298.15 K (Table 1), significantly.

**Table 1:** Gibbs Free Energy of Adsorption (Δ*G*) at 298.15 K and 1 atm in kJ mol^–1^^a^

adsorbate	CLN	CLP	CMOF	BMOF
CH_4_	2.3 to 6.2	–63.6 to −56.0	14.6 to 21.9	17.1 to 20.0
CO_2_	–3.3, 10.6	–68.6	14.4 to 16.3	11.9, 16.6
H_2_	–2.8 to 0.8	–22.5, −22.4	17.0, 17.1	14.2 to 23.8
H_2_O	–46.0 to −36.5	–150.6, −146.1	–8.5, −8.4	–19.2 to 23.2
N_2_	–23.9 to −22.5	–56.1, −55.9	17.6, 17.8	15.0 to 65.7
NO_2_	–107.6 to −88.3	–172.6, −171.4	–54.4 to −31.6	–57.3 to −26.1
O_2_	–98.9 to −73.4	–64.1	–26.2 to −6.9	–3.4 to 22.1
SO_2_	–68.4, −53.2	–135.4 to −134.5	–9.9 to −6.7	4.7 to 18.2

a The min–max value ranges reported indicate several unique adsorption modes. Similarly, single or two discrete values indicate that the geometry or energy of two or several starting adsorption configurations have converged to the same geometry/energy value.

Based on Table 1, at room conditions (298.15 K and 1 atm), only the adsorption of all gases on highly oxidized CLP (or [Fe_4_S_4_]^2+^) clusters is spontaneous. However, when CLP reduces to CLN ([Fe_4_S_4_]^0^), the adsorption efficiency decreases in most cases, particularly with CH_4_ for which the adsorption becomes non-spontaneous. The only exception is O_2_ that adsorbs more favorably on CLN than CLP, increasing the risk of CLN’s oxidative degradation relative to CLP. The comparative oxidative stability of the clusters is discussed in the forthcoming paragraphs.

When the carboxylate and 1,4-benzenedithiolate ligands are added to the CLP cluster, the adsorption free energy increases noticeably. For this reason, BMOF can only adsorb NO_2_ spontaneously and, in some adsorption modes, it can also adsorb H_2_O and O_2_. The high affinity of BMOF in NO_2_ adsorption is because of its capability to initiate Fe–N(NO_2_) interaction and to stabilize the adsorbed NO_2_ molecule through hydrogen bonding between O(NO_2_) and the hydrogen atom of the NH_4_^+^ counter ion (see Figures S1 and S2). On the other hand, CMOF adsorbs more of the gases spontaneously (including H_2_O, NO_2_, O_2_, and SO_2_) but it fails to adsorb CH_4_, CO_2_ H_2_, and N_2_. For CH_4_, CO_2_, and H_2_, only the CLP cluster structure is capable of their efficient adsorption at room temperature, but it would adsorb NO_2_, H_2_O, and SO_2_ more strongly.

In the case of SO_2_, only BMOF rejects its adsorption as predicted by its non-spontaneous adsorption. Also, CMOF should adsorb SO_2_ negligibly, but CLP and CLN adsorb SO_2_ readily through the interactions between the Fe atoms and O atom of SO_2_ (see Figure S1). NO_2_ is the only gas that spontaneously adsorbs onto both MOFs (CMOF and BMOF) and also both clusters (CLP and CLN). Therefore, NO_2_ is the dominant adsorbate on the four CLP, CLN, CMOF, and BMOF adsorbents when all gases are present at the same concentration. Lastly, CMOF and BMOF cannot adsorb N_2_, CH_4_, CO_2_, and H_2_ intrinsically because of lacking effective interactions. Under specific circumstances, such as a high adsorbate concentration, saturation of the MOF pores with these gases will help in their adsorption.

**Figure 2:**
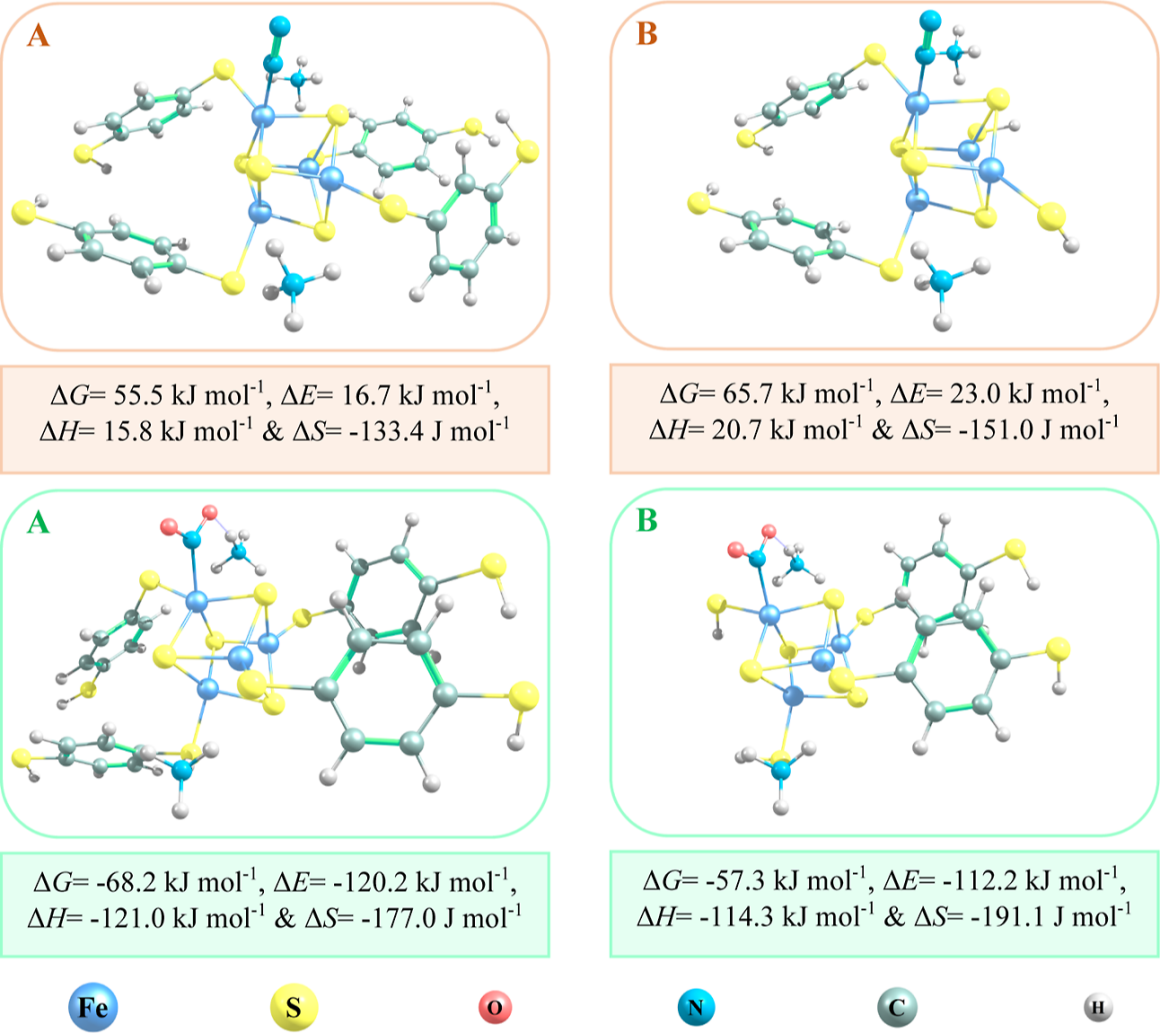
Thermodynamics of N_2_ (orange panels) and NO_2_ (green panels) adsorption on the BMOF models with four (A) and two (B) 1,4-benzenedithiolate ligands.

One important objective is to study whether the increase in the model size affects the thermodynamics results. With the CMOF sorbent, we used a model that contains the required carboxylate ligands and the central metal nodes. However, in the case of the BMOF sorbent, we replaced two of the 1,4-benzenedithiolate ligands with −SH functional groups to decrease the computational cost. Therefore, we selected a secondary BMOF model with four 1,4-benzenedithiolate ligands and replicated the most and least favorable BMOF/adsorbate configurations to evaluate the extent of changes resulting from the inclusion of four organic ligands in the BMOF model. According to Table 1, the least and most favorable gas adsorption configurations are for N_2_ and NO_2_, respectively. Comparing the N_2_/BMOF and NO_2_/BMOF adsorption modes (Figure 2) indicates that changing the model size does not change the adsorbate/BMOF configurations significantly. Consequently, the adsorption energies do not change dramatically. For both adsorbates, the maximum change in energy (about 10 to 11 kJ mol^–1^) is for the Gibbs free energy of adsorption (Δ*G*). As the adsorption energies for both least and most favorable adsorbate/BMOF configurations change coherently, we conclude that the adsorption trends are insensitive to the model size and that the results are qualitatively sufficient to screen the sorbents for different gases in the prospective industrial and environmental applications.

**Figure 3:**
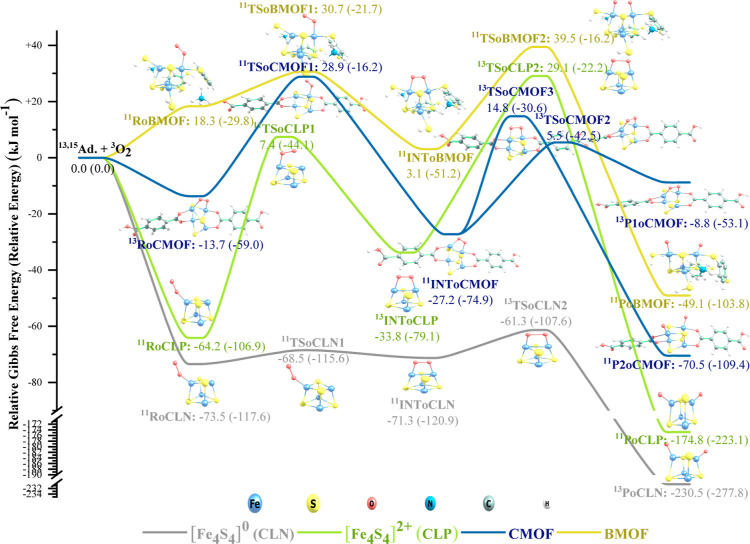
PES for the CLN, CLP, CMOF, and BMOF reactions with molecular oxygen.

**Figure 4:**
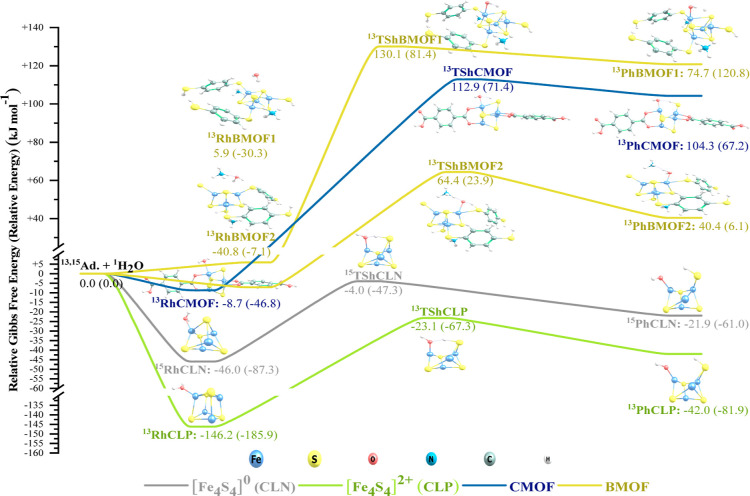
PES for the CLN, CLP, CMOF, and BMOF reactions with water.

Sorbent stability is an important criterion in industrial applications and contributes to their economic viability. Therefore, it is important to study whether the clusters and MOFs are stable enough for practical applications and whether MOF formation increases the stability of [Fe_4_S_4_] clusters. In particular, we focused on the oxidative and hydrolytic stability, and studied the adsorbents’ reaction with ^3^O_2_ and ^1^H_2_O. The obtained reaction profiles or potential energy surfaces (PESs) with energy values and structural transformations are shown in Figures 3 and 4. In these figures, the species along each reaction path are named using their corresponding adsorbent’s name (CMOF, BMOF, CLP, or CLN). Also, the pre-reaction complexes, transition states, reaction intermediates, and products are distinguished, respectively, by including “R”, “TS”, “INT”, and “P” in their names. These labels are followed by “o” for the oxidation paths or “h” for the hydrolysis reactions. The superscript numbers used before the name of each species indicate their spin states.

As shown in Figure 3, the oxidation of the adsorbents starts with the addition of an O_2_ molecule to the [Fe_4_S_4_] cluster. The addition process can be a single-step (observed only for CMOF) or a two-step process. In the two-step mode, first, one oxygen atom adsorbs onto an Fe atom and then the unbound (free) oxygen atom approaches a second Fe atom and bridges over the [Fe_4_S_4_] cluster. After the addition step, the O–O bond starts to stretch, deforming, and eventually breaking up the structure of the [Fe_4_S_4_] cluster. Throughout the oxidation process, electronic surface crossing is possible due to the accessibility of several closely located energy levels, resulting in species with different spin states. In all cases, the oxidized product is more stable than the reactants and the corresponding adsorbate–adsorbent complex before the reaction. These observations agree with the Amitouche et al.^25^ results where dissociative O_2_ chemisorption on [Fe_4_S_4_] was found to be more stable than its non-dissociative physical adsorption. Therefore, neither of the adsorbents is resistant against oxidation from the thermodynamics perspective. However, are they oxidatively stable from the kinetics perspective? We will answer this question after reviewing the hydrolysis mechanism.

In the hydrolysis process (Figure 4), H_2_O adorbs on an Fe atom of the [Fe_4_S_4_] cluster from its oxygen head. Then, one of its H atoms drags toward a neighboring S atom, breaking up the H_2_O molecule to give −SH and an Fe-bound hydroxyl (OH) group. This is similar to the hydrolysis mechanism for the mononuclear FeS cluster of Fe(SCH_3_)_4_^–^ at all its protonation states according to the QM/MM study of Teixeira et al.^13^ For the BMOF sorbent, when the H atom of H_2_O moves toward a cluster’s S atom (i.e., TShBMOF1), the −SH bond does not form, and the H atom stabilizes between the S atom of the cluster and the O atom of H_2_O. The H atom can also transfer to the S atom of the neighboring 1,4-benzenedithiolate ligand (see TShBMOF2 in Figure 4), partially detaching the ligand from the metal node. Simultaneously, the NH_4_^+^ counter ion donates a proton (H^+^) to the Fe-bound OH group, giving the adsorbed H_2_O and NH_3_. Notably, we did not observe a similar ligand detachment for CMOF (the identified transition states could not be approved, see Section S1) but we do not exclude the possibility of ligand hydrolysis for CMOF. Regardless, the hydrolysis reaction is thermodynamically feasible only for the CLN and CLP clusters.

A reaction is kinetically feasible if there is enough energy available to overcome the reaction barriers/transition states to convert the reactants into products. The required energy can be supplied by the surrounding environment or the energy conserved in the vibrational modes of the produced reaction complexes/intermediates. A closer look at the oxidation and hydrolysis reaction profiles (Figures 3 and 4) clarifies that at room conditions (298.15 K and 1 atm), only the hydrolysis of CLN and CLP and the oxidation of CLN are kinetically feasible as their transition states reside below the energy level of the reactants and enough energy can be saved in their intermediate species to pass over the barriers. The oxidation of CLP can also occur, but at a (very) slow rate, because the TSoCLP2 barrier is 29.1 kJ mol^–1^ high relative to the reactants. The other reactions are not feasible without supplying sufficient energy from the environment (i.e., by increasing the temperature). Overall, our results imply that the oxidation of [Fe_4_S_4_]^0^ to [Fe_4_S_4_]^2+^ and also the addition of organic ligands (or MOF formation) can enhance the oxidative and hydrolytic stability of the [Fe_4_S_4_] clusters. Note that the use of 1,4-benzenedithiolate is advantageous over carboxylate in terms of oxidative stability. Our findings agree with the literature, stating that the [Fe_4_S_4_]^2+^ (without bulky ligands) and [Fe_4_S_4_]^0^ clusters are susceptible to oxidative instability.^14,26^

## Conclusions

Our results show that the application of stand-alone [Fe_4_S_4_]^2+^ clusters offers an opportunity to filter out NO_2_, SO_2_, and H_2_O with a low risk of irreversible hydrolysis and oxidative degradation. Therefore, [Fe_4_S_4_]^2+^ clusters are suitable for applications such as atmospheric water harvesting or simultaneous removal of NO_2_ and SO_2_ as two important air pollutants from power plants. These clusters can be also applied to store pure CH_4_, CO_2_, and N_2_ gases or their mixtures, in addition to offering the separation of CH_4_ from H_2_. Furthermore, using [Fe_4_S_4_]^2+^ to design MOFs can noticeably promote their oxidative and hydrolytic stability. The resultant carboxylate and benzenedithiolate MOFs provide high selectivity for removing NO_2_. The carboxylate-containing MOF adsorbs H_2_O and O_2_ negligibly. Neither MOF would adsorb CH_4_, CO_2_, H_2_, or N_2_ at room conditions or at higher temperature levels. Therefore, the benzenedithiolate MOF can be the best option among the studied sorbents for selectively removing NO_2_ from air or flue gas. Moreover, the applications of the MOFs and the CLP cluster can be advantageous in cascade separation processes. Fine-tuning of the [Fe_4_S_4_]-based MOFs and careful adjustment of the process conditions will enhance the applicability of such MOFs to controlled adsorption/desorption processes. Therefore, further experimental and computational studies are recommended. One follow-up study can be the test of the feasibility of O_2_/N_2_ separation using the carboxylate-containing MOF, which has applications in health and energy. Finally, future studies should evaluate the adsorption of N_2_O, which has a significant role in global warming and air pollution.

## Supporting Information Available

Full computational details; adsorption energy, enthalpy, and entropy values; and the most favorable adsorption configurations (PDF)
Cartesian coordinates of the optimized adsorbates, adsorbents, best adsorption configurations, and PES species (ZIP)
