# Supplementary material for: First-Principles Perspective on Gas Adsorption by [Fe4S4]-Based Metal–Organic Frameworks
Source: Langmuir. 2022 Dec 29;39(1):389–94. doi: 10.1021/acs.langmuir.2c02609 (PMC9835974; doi:10.1021/acs.langmuir.2c02609)
Supplement: Supplementary file 1 — la2c02609_si_001.pdf [file la2c02609_si_001.pdf]

Supporting Information for

First-Principles Perspective on Gas Adsorption by [Fe_4S_4]-Based Metal-Organic Frameworks

Fatemeh Keshavarz,[a,*] Nima Rezaei,[b] and Bernardo Barbiellini[a,c]

a) Department of Physics, School of Engineering Science, LUT University, Yliopistonkatu 34, FI-53850 Lappeenranta, Finland.

b) Department of Separation Science, School of Engineering Science, LUT University, Yliopistonkatu 34, FI-53850 Lappeenranta, Finland.

c) Department of Physics, Northeastern University, Boston, MA 02115, USA.

**fatemeh.keshavarz@lut.fi*

Table of Contents

Section S1. Computational details 2

Table S1. Comparison of the [$Fe_4(NO)_4(\mu$-$S)_4$]$^{-1}$ Geometry Optimized at Various Computational Levels and the Doublet Spin State with the Experimentally Resolved Structure 3

Table S2. Comparison of the [$Fe_4S_4Cl_4$]$^{-2}$ Geometry Optimized at Various Computational Levels and the Triplet Spin State with the Experimentally Resolved Structure 4

Table S3. Comparison of the [$Fe_4S_4(SH)_4$]$^{-3}$ Geometry Optimized at Various Computational Levels and the Doublet Spin State with the Experimentally Resolved Structure 4

Table S4. Comparison of the [Fe_4S_4(2,4,6-Trimethyl Benzene Thiolate)$_4$]$^{-2}$ Geometry Optimized at Various Computational Levels and the Triplet Spin State with the Experimentally Resolved Structure 5

Table S5. Electronic Energy of Adsorption (ΔE), in kJ mol^{-1} 8

Table S6. Enthalpy of Adsorption (ΔH) at 298.15 K and 1 atm, in kJ mol^{-1} 8

Table S7. Entropy of Adsorption (ΔS) at 298.15 K and 1 atm, in J mol^{-1} 8

Figure S1. The lowest energy (most favorable) adsorbate/adsorbent configurations 10

REFERENCES 11

Section S1. Computational details

The Gaussian 16 A.03 quantum chemical package[1] was used to study the thermodynamics and kinetics aspects of the studied [Fe_4S_4] clusters and metal-organic framework (MOF) models. Since the Fe atoms in [Fe_4S_4] clusters can be found at different oxidative states and that the cluster might gain an antiferromagnetic state, the quantum chemical treatment of the systems was challenging.[2,3] To improve the accuracy of our results, the computational level was first calibrated using density functional theory (DFT)-based methods and basis sets to study the geometry of several experimentally resolved structures. It should be noted that, from the calibration step to the thermodynamics analysis and the reaction kinetics studies, all potential spin states were evaluated and the one with the minimum zero-point energy (ZPE) electronic energy (and Gibbs free energy) was chosen as the ground state. For computational level calibration, the ground spin state was evaluated at the PBE/def2-TZVP level and then the structures were optimized at various computational levels. For further thermodynamics and kinetics studies, the optimal computational level was used to identify the ground spin state. Also, all calculations included the D3 Grimme's dispersion correction[4] because of the importance of dispersion effects for correct description of the reaction paths and reaction intermediates during the kinetics studies[3] and the potentially weak adsorbate-adsorbent interactions in the thermodynamics evaluations. The only exception was related to the ωB97XD-based calculations, which inherently includes dispersion impacts.[5]

The first compound used in method calibration was [$Fe_4(NO)_4(\mu\text{-}S)_4]^{-1}$. As Table S1 reports, among the selected levels, M06-2X/def2-TZVP reproduces the experimental structure with the highest error (mean absolute error: MAE > 5%). However, PBE/def2-TZVP and PBE/6-311++G** give a significantly more accurate structure with only 0.7% mean absolute error (MAE). Therefore, only these two computational levels were applied to the next test molecules. Comparison of the optimized geometries with the experimental structures in Tables S2-S4 indicates that the two PBE/def2-TZVP and PBE/6-311++G** levels are equally efficient in reproducing the experimental geometries. However, PBE/6-311++G** (average MAE: 1.24%) slightly outperforms PBE/def2-TZVP (average MAE: 1.27%), also featuring a shorter CPU time (see Tables S1 and S2). Therefore, PBE/6-311++G** was selected as the optimal level. This computational level combines the choice of the PBE functional by Amitouche et al.[6] for studying gas adsorption on small Fe-S clusters and improves the choice of B3LYP/6-31++G** by Niu and Ichiye,[7] who stated that the addition of *sp*-type diffuse functions to the 6-31G** basis set enhances the accuracy of the redox energies calculated for [Fe_4S_4] clusters. Notably,

the success of the PBE functional in predicting the FeS cluster properties can be attributed to the cancellation of errors. To concern correlation effect in PBE-based calculations, one can use the DFT + U_{eff} scheme, in which U_{eff} is an orbital-dependent correction to the PBE functional or the Generalized Gradient Approximation (GGA). This parameter is generally expressed as the difference between the Hubbard U parameter, which is the Coulomb energetic cost to place two electrons at the same site, and an approximation of the Hund's exchange parameter J (i.e., $U_{eff} = U - J$). When $U \approx J$, the correlation effect can be ignored and the GGA approach would be adequate. In the case of FeS clusters, U_{eff} is quite small. For example, the U_{eff} of Fe_3S_4 is about 1 eV,[8] and the U and J parameters of pure metallic Fe almost cancel completely.[9] Therefore, the cancellation of the U and J parameters on the iron sites leads to the significant performance of PBE for our studied system.

Table S1. Comparison of the $[Fe_4(NO)_4(\mu\text{-}S)_4]^{-1}$ Geometry Optimized at Various Computational Levels and the Doublet Spin State with the Experimentally Resolved Structure[10]

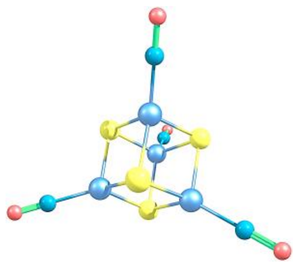

Method	Fe-Fe (Å)	Fe-N (Å)	Fe-S (Å)	N-O (Å)	Fe-S-Fe (°)	S-Fe-S (°)	Fe-N-O (°)	MAE (%)[a]	CPU time (h)[b]
Exp.	2.65	1.66	2.22	1.16	73.4	104.4	177.6	-	-
B3LYP/def2-TZVP	2.87	1.75	2.36	1.17	75.0	103.7	173.4	3.73	284.88
BP86/def2-TZVP	2.64	1.64	2.17	1.19	74.8	103.9	179.3	1.39	281.85
HSE06/def2-TZVP	2.79	1.75	2.33	1.16	74.8	101.6	178.2	2.94	249.53
M06/def2-TZVP	2.80	1.76	2.35	1.16	73.7	103.0	173.2	3.11	195.78
M06-2X/def2-TZVP	3.00	1.82	2.32	1.17	79.2	104.7	178.0	5.23	895.07
M06-L/def2-TZVP	2.70	1.71	2.22	1.17	73.0	106.1	175.1	1.33	299.88
PBE/def2-TZVP	2.63	1.65	2.21	1.18	73.1	104.3	179.2	0.71	191.25
PBE0/def2-TZVP	2.84	1.75	2.34	1.16	74.7	103.9	173.2	3.25	278.80
wB97X-D/def2-TZVP	2.80	1.75	2.26	1.16	73.5	104.7	177.9	1.93	272.89
PBE/6-31G	2.72	1.66	2.39	1.22	71.2	102.6	172.4	3.30	21.85
PBE/6-31+G*	2.63	1.65	2.22	1.20	72.8	104.7	179.2	0.97	191.22
PBE/6-311++G**	2.65	1.65	2.22	1.19	73.0	104.8	178.9	0.69	153.14
PBE/cc-pVDZ	2.66	1.64	2.24	1.19	73.4	104.3	178.7	0.83	302.05

[a] MAE: Mean absolute error

[b] Total CPU time for full optimization of the structure and frequency calculation

Table S2. Comparison of the $[Fe_4S_4Cl_4]^{-2}$ Geometry Optimized at Various Computational Levels and the Triplet Spin State with the Experimentally Resolved Structure[11]

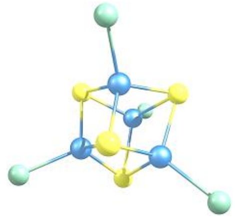

	Fe–S (Å)	Fe–Cl (Å)	Fe–S–Fe (°)	S–Fe–S (°)	S–Fe–Cl (°)	MAE (%)[a]	CPU time (hr)[b]
Exp.	2.24–2.31	2.20–2.24	72.9–74.9	102.7–105.4	109.6–120.2	-	-
PBE/6-311++G**	2.23–2.31	2.24–2.25	69.9–73.77	101.86–104.75	110.79–116.97		
(Error %)[a]	(0.22)	(1.13)	(2.84)	(0.72)	(0.87)	1.16	94.34
PBE/def2-TZVP	2.21–2.30	2.23–2.24	70.7–74.1	101.8–108.0	111.0–117.2		
(Error %)	(0.88)	(0.68)	(2.03)	(0.82)	(0.70)	1.02	372.27

[a] Error and MAE (Mean absolute error) calculated according to the median experimental and theoretical values
[b] Total CPU time for full optimization of the structure and frequency calculation

Table S3. Comparison of the $[Fe_4S_4(SH)_4]^{-3}$ Geometry Optimized at Various Computational Levels and the Doublet Spin State with the Experimentally Resolved Structure[12 a]

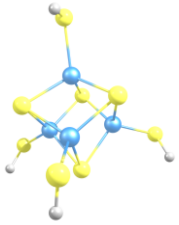

	Fe-Sc (Å)	Fe-Fe (Å)	S_c-Fe-S_c (°)	Sc-Fe-S_t (°)	Fe-S-Fe (°)	MAE (%)[b]
Exp.	2.3100	2.7600	104.3	114.2	73.5	-
PBE/6-311++G**	2.31	2.76	104.3	114.3	73.1	
(Error %)	(0.00)	(0.00)	(0.00)	(0.09)	(0.54)	0.13
PBE/def2-TZVP	2.30	2.77	104.0	114.4	74.0	
(Error %)	(0.43)	(0.36)	(0.29)	(0.18)	(0.68)	0.39

[a] All reported experimental and theoretical values are average values. S_c: Sulfur atom of the cubane cluster; and S_t: Sulfur atom of the SH group.
[b] MAE: Mean absolute error

Table S4. Comparison of the $[Fe_4S_4(2,4,6\text{-Trimethyl Benzene Thiolate})_4]^{-2}$ Geometry Optimized at Various Computational Levels and the Triplet Spin State with the Experimentally Resolved Structure[13] [a]

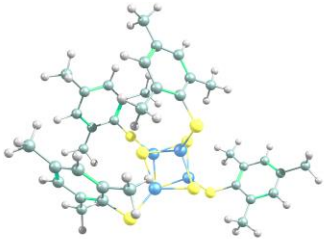

	Fe-Fe (Å)	Fe-S_t (Å)	Fe-S_c (Å)	S_t-C (Å)	S_t-Fe-S_c (°)	Fe-S_c-Fe (°)	S_c-Fe-Sc (°)	Fe-S_t-C (°)	MAE (%)[a]
Exp.	2.74–2.80	2.27–2.28	2.27–2.31	1.77–1.79	111.8–119.8	74.4 (avg)[c]	103.8 (avg)	99.4–100.4	-
PBE/6-311++G**	2.58–2.63	2.22–2.27	2.14–2.23	1.79	107.4–117.7	72.4 (avg)	104.2 (avg)	102.6–108.5	
(Error %)[a]	(5.96)	(1.32)	(4.59)	(0.56)	(2.81)	(2.69)	(0.39)	(5.71)	3.00
PBE/def2-TZVP	2.58–2.62	2.21–2.26	2.12–2.24	1.78	107.9–119.6	72.85 (avg)	104.4 (avg)	104.2–108.2	
(Error %)	(6.14)	(1.76)	(5.24)	(0.00)	(1.77)	(2.08)	(0.58)	(6.31)	2.98

[a] S_c: Sulfur atom of the cubane cluster; and S_t: Sulfur atom of the thiolate ligand
[b] Error and MAE (Mean absolute error) calculated according to the median/average experimental and theoretical values
[c] avg: average value reported

After computational level calibration, we selected our $[Fe_4S_4]$ clusters of interest. It is known that the $[Fe_4S_4]^0$ and $[Fe_4S_4]^{1+}$ oxidation states are not as achievable as the $[Fe_4S_4]^{2+}$ state,[14] and the $[Fe_4S_4]^{1+}$ and $[Fe_4S_4]^{2+}$ states are more stable.[15] Therefore, we focused on the least ($[Fe_4S_4]^0$) and most ($[Fe_4S_4]^{2+}$) stable oxidation states. For the MOF models, we used $[Fe_4S_4]^{2+}$ metal nodes because that is the most commonly reported oxidation state for ligand bearing $[Fe_4S_4]$ clusters.[2,11,15,16]

The next concern was the spin state of the clusters. Many studies have stated that the presence of thiolates and sulfates can induce weak ligand field splitting, resulting in high spin states for the Fe^{2+}/Fe^{3+} ions. As a consequence, the unpaired electrons of the metal ions can interact to give a variety of ferro- and antiferromagnetic states.[2] In the case of biosystems, $[Fe_4S_4]^{2+}$ clusters usually split into two high spin planes that give the total spin of 0 (antiferromagnetic state).[2,17,18] As implied, the microenvironment (ligands) surrounding the cluster can affect the total spin state of the systems. Therefore, we screened all potential ferro- and antiferromagnetic spin states. For the antiferromagnetic state, we applied the recommended spin unrestricted broken symmetry calculations[2,17,19-21] by splitting the cluster structure into two Fe_2S_2 fragments, assigning the highest spin state to each fragment while keeping the total spin of the system equal to zero. After energy evaluations and checking the possibility of structures having imaginary potential frequencies or spin contamination, we concluded that the ferromagnetic

states of our isolated [Fe_4S_4] clusters and the MOF models are more stable compared to the antiferromagnetic state. The most stable spin state for $[Fe_4S_4]^{2+}$ (called CLP hereafter) and the MOF models was found to be 13tet, while the $[Fe_4S_4]^0$ cluster (CLN) was found to be 15tet. As an example, 13tet CLP was found 59.5 kJ mol^{-1} more stable than its singlet antiferromagnetic state, at 0 K. Furthermore, the difference in their Gibbs free energy at 298.15 K and 1 atm was calculated to be 82.5 kJ mol^{-1}. Therefore, we skipped the antiferromagnetic state in our thermodynamics and kinetics studies, but still screened all potential ferromagnetic spin states when the presence of the adsorbate could alter the spin state of the system (such as in $^3O_2$ and $^2NO_2$ adsorption).

After calibrating all basic quantum chemical parameters, we started the thermodynamics and kinetics calculations. For the thermodynamic analysis, we placed the adsorbate molecules (one at a time) around each cluster/MOF adsorbent at different configurations and optimized their geometries and electronic structures. The adsorbate molecules were added in their most stable spin state; $^1CH_4$, $^1CO_2$, ^1H_2O, $^1N_2$, $^2NO_2$, $^3O_2$, and $^1SO_2$. After optimization, the structures giving imaginary frequencies were eliminated as unstable adsorbate/adsorbent pairs. Also, many starting configurations led to identical adsorbate/adsorbent final structures. Non-unique structures were removed from the final results. The default harmonic frequencies and rigid-rotor approximations were used to calculate the partition functions and thermodynamic parameters of each structure. Using the final thermodynamics parameters, the adsorption energy (ΔE), enthalpy (ΔH), entropy (ΔS) and Gibbs free energy (ΔG) values were calculated as the energy difference between the energy of the adsorbate/adsorbent system and that of the isolated adsorbate and adsorbent molecules. For example, the Gibbs free energy of adsorption was calculated based on the following equation:

$$\Delta G_{i,j} = G_{adsorbate\ i/adsorbent\ j} - G_{adsorbate\ i} - G_{adsorbent\ j}$$

where $\Delta G_{i,j}$ is the adsorption Gibbs free energy of adsorbate i adsorbed on adsorbent j, and $G_{adsorbate\ i}$ and $G_{adsorbent\ j}$ are respectively the Gibbs free energy of the isolated adsorbate i and adsorbent j molecules/clusters.

In addition to the thermodynamics analysis, we estimated the oxidative and hydrolytic stability of the clusters and MOF models by studying their reaction with $^3O_2$ and ^1H_2O, respectively. First, a number of different reaction transition states (TSs) were guessed and optimized. If the optimized structure associated with one imaginary frequency, intrinsic reaction coordinate (IRC) analysis[22,23] was performed to connect that TS to the related product(s) and reactant(s). Often, the proposed TSs did not lead to any product/reactant and were neglected. The identified reactants/products were optimized and accepted as reactants, products, or reaction

intermediates if they lacked any imaginary frequencies. Then, the confirmed reaction paths were combined to construct the potential energy surfaces (PESs).

Table S5. Electronic Energy of Adsorption (ΔE), in kJ mol^{-1} [a]

Adsorbate	CLN	CLP	CMOF	BMOF
CH_4	-33.8 to -28.7	-101.6 to -98.1	-11.4 to -10.5	-15.4 to -11.8
CO_2	-36.5, -22.6	-105.8	-16.8 to -14.3	-24.1, -14.4
H_2	-28.5 to -26.4	-49.4, -49.3	-3.6	-5.7 to 5.9
H_2O	-87.3, -73.8	-185.5, -185.8	-46.5	-60.2 to -18.8
N_2	-63.9, -57.3	-91.2	-10.5	-19.6 to 23.0
NO_2	-159.2 to -135.9	-214.4, -213.9	-101.7 to -79.3	-112.2 to -86.9
O_2	-146.1 to -117.5	-106.9	-72.2, -49.4	-49.5 to -5.8
SO_2	-107.2, -100.2	-178.5, -175.9	-51.6, -49.8	-49.7 to -27.4

[a] The min-max value ranges reported indicate several unique adsorption modes. Similarly, single or two discrete values indicate that the geometry or energy of two or several starting adsorption configurations have converged to the same geometry/energy value. The green cells indicate favorable adsorption, and the blue cell shows that adsorption favorability alters with adsorption mode.

Table S6. Enthalpy of Adsorption (ΔH) at 298.15 K and 1 atm, in kJ mol^{-1} [a]

Adsorbate	CLN	CLP	CMOF	BMOF
CH_4	-32.3, -27.4	-101.8, -101.0	-8.4, -8.3	-13.5 to -8.8
CO_2	-34.1, -20.4	-105.4, -105.3	-14.3, -11.6	-22.3, -11.8
H_2	-33.5 to -29.0	-54.7 to -54.6	-4.1	-6.4 to 5.8
H_2O	-89.7, -75.2	-189.1, -188.8	-47.9, -47.8	-64.2 to -20.8
N_2	-65.2, -57.0	-92.3	-8.6	-18.5 to 20.7
NO_2	-160.2 to -135.2	-213.0, -212.5	-102.1 to -78.9	-114.3 to -90.6
O_2	-148.5, -119.4	-109.3	-74.9 to -50.2	-52.9 to -3.9
SO_2	-105.2, -98.9	-177.5, -174.5	-51.7 to -47.2	-50.5 to -25.8

[a] The min-max value ranges reported indicate several unique adsorption modes. Similarly, single or two discrete values indicate that the geometry or energy of two or several starting adsorption configurations have converged to the same geometry/energy value.

Table S7. Entropy of Adsorption (ΔS) at 298.15 K and 1 atm, in J mol^{-1} [a]

Adsorbate	CLN	CLP	CMOF	BMOF
CH_4	-117.1 to -112.8	-132.1 to -127.9	-90.6 to -76.8	-112.2 to -86.8
CO_2	-104.1 to -103.2	-123.8, -123.3	-96.1 to -93.3	-114.7 to -95.1
H_2	-115.1 to -88.0	-108.5 to -107.6	-71.0, -70.7	-86.6 to -59.3
H_2O	-146.7 to -129.9	-144.3, -127.9	-132.3, -132.1	-156.1 to -129.5
N_2	-138.9 to -115.8	-121.9 to -121.2	-88.3 to -87.7	-151.4 to -77.4
NO_2	-176.6 to -157.3	-139.6, -133.8	-166.0 to -158.6	-216.1 to -191.1
O_2	-166.5, -154.2	-151.7	-163.7 to -144.8	-172.5 to -87.2
SO_2	-153.3, -123.3	-141.1 to -133.7	-132.0 to -129.2	-185.1 to -134.4

[a] The min-max value ranges reported indicate several unique adsorption modes. Similarly, single or two discrete values indicate that the geometry or energy of two or several starting adsorption configurations have converged to the same geometry/energy value.

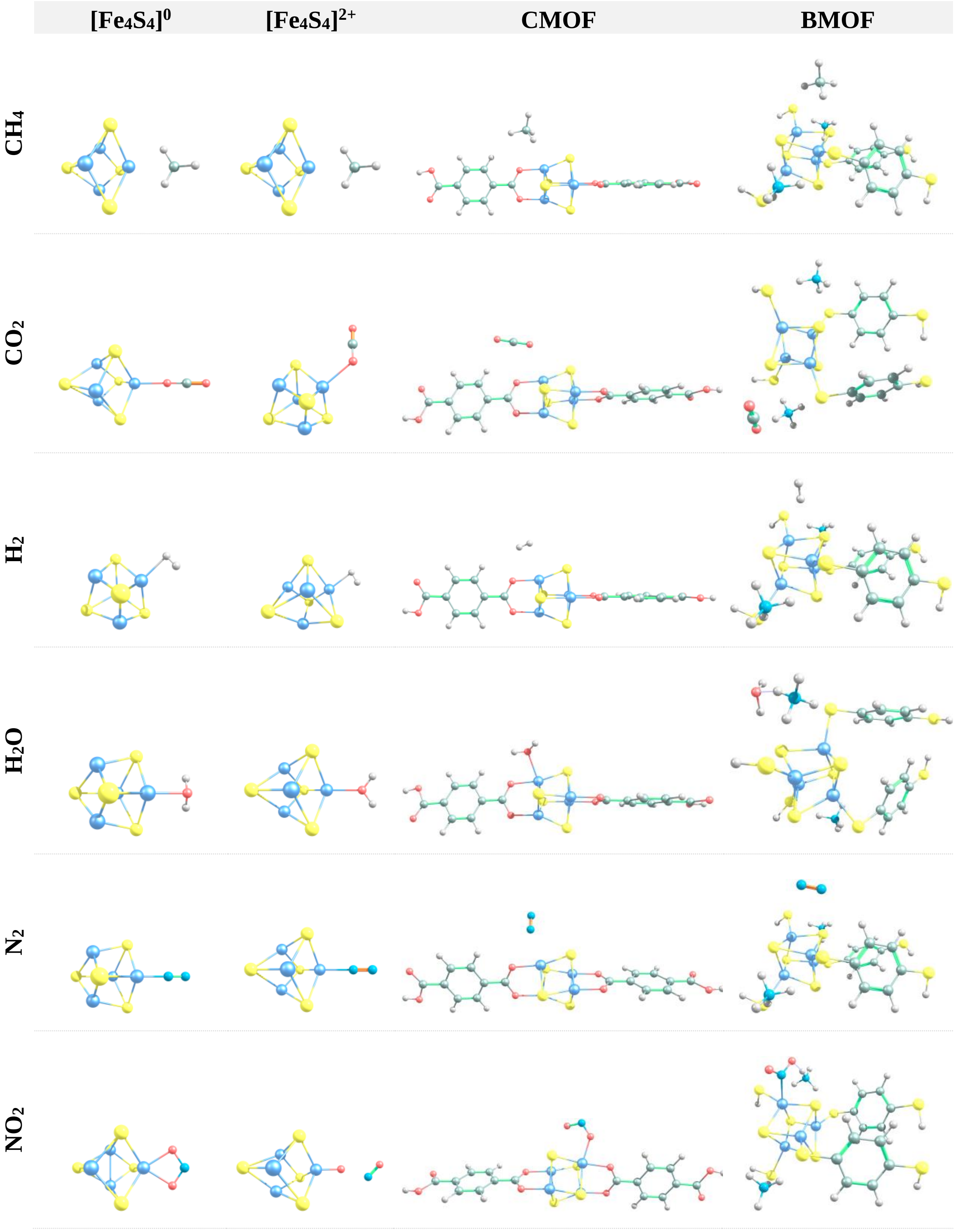
[Fe4S4]0
[Fe4S4]2+
CMOF
BMOF
CH4
CO2
H2
H2O
N2
NO2

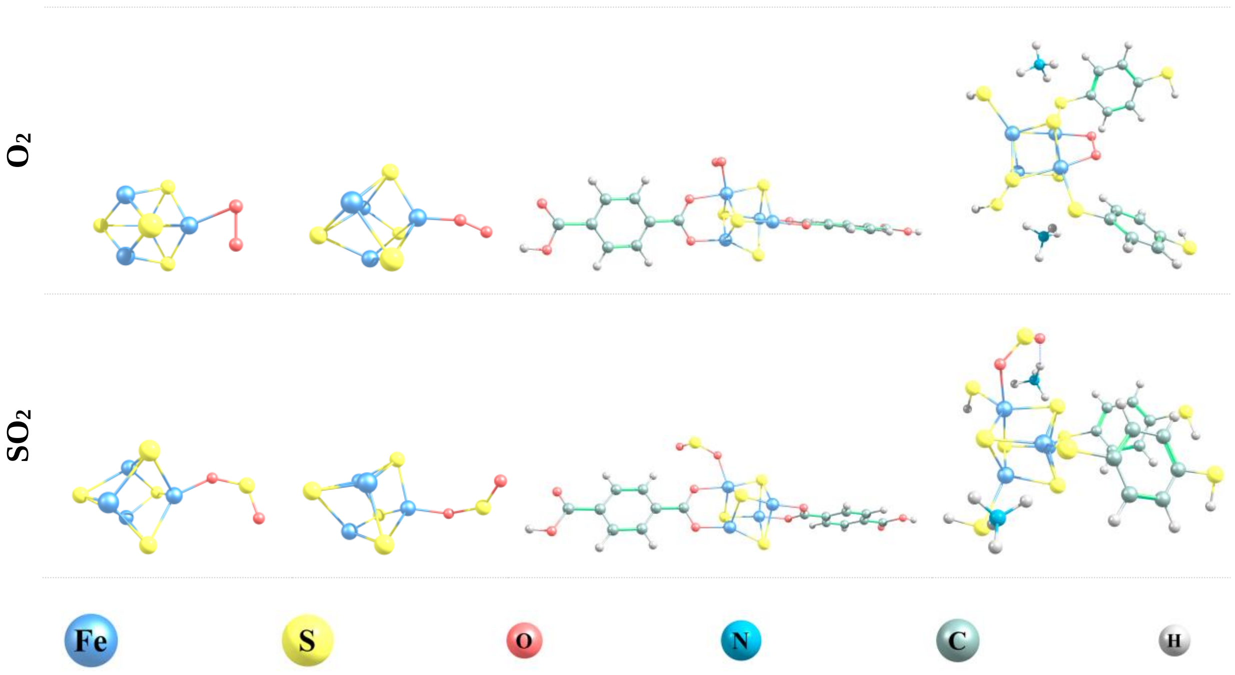

Figure S1. The lowest energy (most favorable) adsorbate/adsorbent configurations.

REFERENCES

(1) Frisch, M. J.; Trucks, G. W.; Schlegel, H. B.; Scuseria, G. E.; Robb, M. A.; Cheeseman, J. R.; Scalmani, G.; Barone, V.; Petersson, G. A.; Nakatsuji, H.; et al. Gaussian 16, Revision A.03, Gaussian, Inc., Wallingford CT, 2016.

(2) Szilagyi, R. K.; Winslow, M. A. On the Accuracy of Density Functional Theory for Iron—Sulfur Clusters. *J. Comput. Chem.* **2006**, *27*, 1385-1397.

(3) Teixeira, M. H.; Curtolo, F.; Camilo, S. R. G.; Field, M. J.; Zheng, P.; Li, H.; Arantes, G. M. Modeling the Hydrolysis of Iron–Sulfur Clusters. *J. Chem. Inf. Model.* **2019**, *60*, 653-660.

(4) Grimme, S.; Antony, J.; Ehrlich, S.; Krieg, H. A Consistent and Accurate Ab initio Parameterization of Density Functional Dispersion Correction (DFT-D) for the 94 Elements H-Pu. *J. Chem. Phys*. **2010**, *32*, 154104

(5) Chai, J.-D.; Head-Gordon, M. Long-Range Corrected Hybrid Density Functionals with Damped Atom-Atom Dispersion Corrections. *Phys. Chem. Chem. Phys.* **2008**, *10*, 6615-6620.

(6) Amitouche, F.; Saad, F.; Tazibt, S.; Bouarab, S.; Vega, A. Structural and Electronic Rearrangements in Fe_2S_2, Fe_3S_4, and Fe_4S_4 Atomic Clusters under the Attack of NO, CO, and O_2. *J. Phys. Chem. A* **2019**, *123*, 10919-10929.

(7) Niu, S.; Ichiye, T. Cleavage of [4Fe-4S]-Type Clusters: Breaking the Symmetry. *J. Phys. Chem. A* **2009**, *113*, 5710-5717.

(8) Devey, A. J.; Grau-Crespo, R.; De Leeuw, N. H. Electronic and Magnetic Structure of Fe_3S_4: GGA+ U Investigation. *Phys. Rev. B* **2009**, *79*, 195126.

(9) Yang, I.; Savrasov, S. Y.; Kotliar, G. Importance of Correlation Effects on Magnetic Anisotropy in Fe and Ni. *Phys. Rev. Lett.* **2001**, *87*, 216405.

(10) Ting-Wah Chu, C.; Yip-Kwai Lo, F.; Dahl, L. F. Synthesis and Stereochemical Analysis of the $[Fe_4(NO)_4(\mu_3\text{-}S)_4]_n$ series (n= 0,-1) which Possesses a Cubanelike Fe4S4 Core: Direct Evidence for the Antibonding Tetrametal Character of the Unpaired Electron upon a One-Electron Reduction of a Completely Bonding Tetrahedral Metal Cluster. *J. Am. Chem. Soc.* **1982**, *104*, 3409-3422.

(11) Al-Rammahi, T. M. M.; Waddell, P. G.; Henderson, R. A. X-ray Crystal Structures of $[NHR_3]_2[Fe_4S_4X_4]$ (X= PhS, R= Et or n Bu; X= Cl, R= n Bu): Implications for Sites of Protonation in Fe–S Clusters. *Transit. Met. Chem.* **2016**, *41*, 555-561.

(12) Segal, B. M.; Hoveyda, H. R.; Holm, R. H. Terminal Ligand Assignments Based on Trends in Metal−Ligand Bond Lengths of Cubane-Type $[Fe_4S_4]^{2+,+}$ Clusters. *Inorg. Chem.* **1998**, *37*, 3440-3443.

(13) Ueyama, N.; Sugawara, T.; Fuji, M.; Nakamura, A.; Yasuoka. N. Crystal Structure of $[Et_4N]_2[Fe_4S_4(2, 4, 6\text{-Trimethylbenzenethiolato})_4]$. *Chem. Lett.* **1985**, *14*, 175-178.
(14) Deng, L.; Majumdar, A.; Lo, W.; Holm, R. H. Stabilization of 3:1 Site-Differentiated Cubane-Type Clusters in the $[Fe_4S_4]^{1+}$ Core Oxidation State by Tertiary Phosphine Ligation: Synthesis, Core Structural Diversity, and S= 1/2 Around States. *Inorg. Chem.* **2010**, *49*, 11118-11126.
(15) Horwitz, N. E.; Xie, J.; Filatov, A. S.; Papoular, R. J.; Shepard, W. E.; Zee, D. Z.; Grahn, M. P.; Gilder, C.; Anderson, J. S. Redox-Active 1D Coordination Polymers of Iron–Sulfur Clusters. *J. Am. Chem. Soc.* **2019**, *141*, 3940-3951.
(16) Liu, Q.; Zhang, C.; Chen, C.; Zhu, H.; Deng, Y.; Cai, J. Syntheses and Structural Characterizations of Fe_4S_4 Cubane-like Cluster Compounds Containing Cycloalkylthiolate Ligands. *Sci. China, Ser. B: Chem.* **1997**, *40*, 616-623.
(17) Torres, R. A.; Lovell, T.; Noodleman, L.; Case, D. A. Density Functional and Reduction Potential Calculations of Fe_4S_4 Clusters. *J. Am. Chem. Soc.* **2003**, *125*, 1923-1936.
(18) Kubas, A.; Maszota, P. Theoretical Insights into the Unique Ligation of $[Fe_4S_4]$ Iron–Sulfur Clusters. *Eur. J. Inorg. Chem.* **2018**, *2018*, 2419-2428.
(19) Shoji, M.; Koizumi, K.; Kitagawa, Y.; Yamanaka, S.; Kawakami, T.; Okumura, M.; Yamaguchi, K. Theory of Chemical Bonds in Metalloenzymes II: Hybrid-DFT Studies in Iron–Sulfur Clusters. *Int. J. Quantum Chem.* **2005**, *105*, 628-644.
(20) Dey, A.; Glaser, T.; Couture, M. M.-J.; Eltis, L. D.; Holm, R. H.; Hedman, B.; Hodgson, K. O.; Solomon, E. I. Ligand K-Edge X-ray Absorption Spectroscopy of $[Fe_4S_4]^{1+,2+,3+}$ Clusters: Changes in Bonding and Electronic Relaxation upon Redox." *J. Am. Chem. Soc.* **2004**, *126*, 8320-8328.
(21) Bruschi, M., Greco, C.; Fantucci, P.; Gioia, L. D. Structural and Electronic Properties of the [FeFe] Hydrogenase H-Cluster in Different Redox and Protonation States. A DFT Investigation. *Inorg. Chem.* **2008**, *47*, 6056-6071.
(22) Fukui, K. The Path of Chemical-Reactions – The IRC Approach. *Acc. Chem. Res.* **1981**, *14*, 363-368.
(23) Hratchian, H. P.; Schlegel, H. B. *Theory and Applications of Computational Chemistry: The First 40 Years*, Elsevier, Amsterdam, 2005, 195-249.
